# Psychological responses and lifestyle changes among pregnant women with respect to the early stages of COVID-19 pandemic

**DOI:** 10.1177/0020764020952116

**Published:** 2020-08-20

**Authors:** Yingfei Zhang, Zheng Feei Ma

**Affiliations:** 1 Mathematics Teaching and Research Office, Public Basic College, Jinzhou Medical University, Jinzhou, Liaoning, China; 2 Department of Health and Environmental Sciences, Xi’an Jiaotong-Liverpool University, Suzhou, China

**Keywords:** Stress, mental health, IES, pregnant women, China

## Abstract

**Objectives:**

The COVID-19 pandemic has caused a profound impact on health and well-being of populations. However, there are limited studies that have investigated the psychological aspects of vulnerable groups including pregnant women amid the COVID-19 pandemic. Therefore, we aimed to assess the psychological impact of the COVID-19 pandemic among Chinese pregnant women from February 2020 until March 2020.

**Methods:**

Our study was conducted using a modified validated online questionnaire comprising of sociodemographic, the Impact of Event Scale (IES), attitude and mental health-related questions towards COVID-19.

**Results:**

A total of 560 women were included. The overall mean age and IES of women was 25.8 ± 2.7 years and 31.4 ± 13.7. Moreover, 67.1% of them had IES ⩾26. Psychological impact seemed to be more severe in women in second trimester of pregnancy (the highest IES) (*p* = .016). There was a significant association between trimesters of pregnancy and some indicators of negative health impacts (including increased stress from work, increased stress from home, feeling apprehensive and helpless during the early stages of the COVID-19 pandemic) (all *p* < .05).

**Conclusions:**

Our results reported moderate-to-severe stressful impact among Chinese pregnant women. We recommend that appropriate measures should be taken to address the maternal mental health issues.

## Introduction

China has been deeply affected by the coronavirus disease 2019 (COVID-19) pandemic which was caused by severe acute respiratory syndrome coronavirus 2 (SARS-CoV-2) since December 2019. COVID-19 has been considered one of the most serious public health emergencies in the 21st century, which has severely affected the economy, public health and quality of life of different population groups globally (El-Zoghby et al., 2020; Ma et al., 2020; Qiu et al., 2020; Zhang & Ma, 2020).

In the early stage of the COVID-19 pandemic, some studies have reported increased prevalence and severity of mental health-related problems in the general populations (El-Zoghby et al., 2020; Ma et al., 2020; Qiu et al., 2020; Zhang & Ma, 2020). Most of the concerns about the psychological impacts regarding the COVID-19 pandemic are conducted among the general populations (El-Zoghby et al., 2020; Ma et al., 2020; Qiu et al., 2020; Zhang & Ma, 2020). In addition, the implementation of intensive health precautions including quarantine wearing masks and social distancing may have also exacerbate the severity of stress impact, especially in the vulnerable populations including pregnant women (Saccone et al., 2020).

Pregnant women are considered a uniquely vulnerable group because of their compromised immunological functions, altered physiology and susceptibility to infections (Dashraath et al., 2020). Pregnant women may experience stress, anxiety and depression associated with some potential adverse obstetrical outcomes including foetal death. In addition, pregnant women may encounter increased stress, anxiety and depression levels during infectious disease outbreaks such as the current COVID-19 pandemic. However, these impacts of the COVID-19 pandemic, both positive and negative, have not been extensively assessed in pregnant women, especially in developing countries such as China.

Therefore, the aim of the present study was to assess the attitude towards COVID-19, psychological and stress impact among pregnant women amid the COVID-19 pandemic’s immediate wake.

## Methods

A cross-sectional study was conducted online via WeChat (a Chinese social media platform) between February and March 2020 to recruit a convenience sample of pregnant women residing in Liaoning Province, China which is located in the Northeast region of China. Since it was difficult to recruit pregnant women, snowball sampling was used. Only pregnant women of Chinese nationality aged ⩾18 years and willing to give informed consent were recruited into the study. The study protocol was reviewed and approved by the Ethics Committee of the Jinzhou Medical University (ref. no. JYDLL2020002). In addition, the study was performed in accordance with The Code of Ethics of the World Medical Association (Declaration of Helsinki).

### Impact of event scale

A modified validated IES questionnaire in Chinese language with a Cronbach’s alpha of .8 was used to measure the psychological impact amid the COVID-19 pandemic (Ma et al., 2020; Zhang & Ma, 2020). The IES questionnaire comprised of two subscales (i.e. intrusive and avoidance). All items in the IES questionnaire have a response set ranging from 0 (for not all) to 5 (for often). An overall IES score of ⩾26 was used to indicate moderate-to-severe psychological impact experienced by pregnant women amid the COVID-19 pandemic.

### Family and social support

Questions regarding family and social support received amid the COVID-19 pandemic with a Cronbach’s alpha of .8 were included: support received from family members, caring for family members’ feeling, sharing of feeling with other family members, sharing of feeling with others and support received from friends (Ma et al., 2020; Zhang & Ma, 2020). Each question was based on a response of 5-point Likert scale ranging from 1 (for much decreased) to 5 (for much increased). A lower score received by pregnant women was used to suggest limited family and social support amid the COVID-19 pandemic (Ma et al., 2020; Zhang & Ma, 2020).

### Mental health-related lifestyle changes

In addition, a 4-item questionnaire assessing the mental health-related lifestyle changes amid the COVID-19 pandemic with a Cronbach’s alpha value of .8 using the Mental Health Lifestyle Scale (MHLSS) was included in the study (Ma et al., 2020; Zhang & Ma, 2020). Each question evaluated the degree to which pregnant women felt that they have changes their mental health-related lifestyles using a 5-point Likert scale ranging from 1 (much decreased) to 5 (much increased). A lower score in MHLSS was used to suggest a less positive changes in their mental health-related lifestyle amid the COVID-19 pandemic (Lau et al., 2006; Zhang & Ma, 2020).

### Attitudes towards COVID-19 and indicators of negative mental health impacts

Participants were also asked to complete questions regarding their attitudes towards the COVID-19 pandemic with a Cronbach’s alpha value of .9. These questions included: ‘know SARS-CoV-2 and relevant prevention knowledge well’, ‘concerned about the COVID-19 pandemic’, ‘the COVID-19 pandemic is far away from me’ and ‘pregnant women are more vulnerable to the COVID-19 than other non-pregnant population groups’. In addition, questions regarding the negative mental health impacts amid the COVID-19 pandemic with a Cronbach’s alpha of .88 (Ma et al., 2020; Zhang & Ma, 2020). These questions assessed the changes of stress from work, home, financial stress, helpless feeling due to the COVID-19 pandemic, horrified feeling due to the COVID-19 pandemic and apprehensive feeling due to the COVID-19 pandemic, with response options ranging from 1 (much decreased) to 5 (much increased) (Ma et al., 2020; Zhang & Ma, 2020).

### Statistical analysis

Statistical analysis was conducted using SPSS ver. 24.0 (IBM Corp., Armonk, NY, USA). A Cronbach’s alpha was calculated to assess the reliability of the questionnaires used in the study. Continuous and categorical data were expressed as mean ± standard deviation and number (%), respectively. Chi-square tests were used to compare the differences between categorical data. Analyses of covariance (ANCOVA) tests adjusting for age were employed to determine the differences between continuous data. Regression models were used to examine the predictors of IES in pregnant women amid the COVID-19 pandemic. A two-tailed *p*-value <.05 was used to denote statistical significance.

## Results

### Participant characteristics

Of 600 pregnant women who were invited to complete the questionnaire, 560 pregnant women were included in the final analysis with a completion rate of 93.3% (Table 1). Those who declined the study invitation explained that they were not interested to participate (*n* = 40). The mean age of pregnant women was 25.8 ± 2.7 years, with more than half of pregnant women (79.8%) were in first and second trimesters of pregnancy. Majority of pregnant women reported having earned a higher educational degree (92.0%) and self-identified as not having any religious beliefs (96.6%). None of the pregnant women were positive for the COVID-19 at the time of the study.

**Table 1.** Sociodemographic characteristics of pregnant women by trimesters of pregnancy.

	All (*n* = 560)	Trimesters			*p*-value
		First (*n* = 227)	Second (*n* = 220)	Third (*n* = 113)	
Age (years)	25.8 ± 2.7	25.3 ± 2.8	26.4 ± 2.0	25.7 ± 3.3	<.001
Education level, *n* (%)					
*Secondary school*	39 (8.0)	16 (7.0)	14 (6.4)	9 (8.0)	.851
*Higher qualification*	103 (92.0)	211 (93.0)	206 (93.6)	103 (92.0)	
Religion, *n* (%)					
*No*	541 (96.6)	220 (96.9)	214 (97.3)	107 (94.7)	.374
*Yes*	19 (3.4)	7 (3.1)	6 (2.7)	6 (5.3)	

### Impact on family and social support

Majority of pregnant women reported increased support from their friends (75.2%), family members (85.9%), caring for their family members (81.2%), shared feeling with their family members (83.2%) and others when feeling blue (89.6%) during the early stages of the COVID-19 pandemic (Table 2). Pregnant women in second and third trimesters of pregnancy were more likely to receive support from family members and care for family members’ feelings than pregnant women in first trimester of pregnancy (all *p* < .05).

**Table 2.** Changes in family and social support by trimesters of pregnancy.

	Trimesters			*p*-value
	First (*n* = 227)	Second (*n* = 220)	Third (*n* = 113)	
Getting support from friends, *n* (%)				
*Decreased*	11 (4.8)	2 (0.9)	1 (0.9)	.065
*Same as before*	51 (22.5)	50 (22.7)	24 (21.2)	
*Increased*	165 (72.7)	168 (76.4)	88 (77.9)	
Getting support from family members, *n* (%)				
*Decreased*	11 (4.8)	2 (0.9)	1 (0.9)	.003
*Same as before*	36 (15.9)	19 (8.6)	10 (8.8)	
*Increased*	180 (79.3)	199 (90.5)	102 (90.3)	
Shared feeling with family members, *n* (%)				
*Decreased*	7 (3.1)	2 (0.9)	2 (1.8)	.075
*Same as before*	47 (20.7)	21 (9.5)	15 (13.3)	
*Increased*	173 (76.2)	197 (89.5)	96 (85.0)	
Shared feeling with others when feeling blue, *n* (%)				
*Decreased*	9 (4.0)	3 (1.4)	4 (3.5)	.230
*Same as before*	20 (8.8)	17 (7.7)	5 (4.4)	
*Increased*	198 (87.2)	200 (90.9)	104 (92.0)	
Caring for family members’ feelings, *n* (%)				
*Decreased*	6 (2.6)	2 (0.9)	6 (5.3)	.035
*Same as before*	46 (20.3)	30 (13.6)	15 (13.3)	
*Increased*	175 (77.1)	188 (85.5)	92 (81.4)	

### Impact on mental health-related lifestyle changes

During the early stages of the COVID-19 pandemic, majority of pregnant women reported that they had paid more attention to their mental health condition (85.9%), spent time to rest (71.4%) and relax (73.4%). On the other hand, more than half of pregnant women (78.0%) reported that the time spent to exercise as before the COVID-19 outbreak (Table 3). Pregnant women in third trimester of pregnancy were more likely to pay more attention to mental health than pregnant women in second and third trimester of pregnancy (*p* = .011). In addition, pregnant women in second and third trimesters of pregnancy were more likely to spend more time to rest and relax than pregnant women in first trimester of pregnancy (all *p* < .05). However, pregnant women in first trimester of pregnancy were more likely to spend more time exercising than pregnant women in second and third trimesters of pregnancy (*p* < .001).

**Table 3.** Awareness and lifestyles by trimesters of pregnancy.

	Trimesters			*p*-value
	First (*n* = 227)	Second (*n* = 220)	Third (*n* = 113)	
Pay attention to mental health, *n* (%)				
*Decreased*	6 (2.6)	2 (0.9)	0 (0.0)	.011
*Same as before*	35 (15.4)	30 (13.6)	6 (5.3)	
*Increased*	186 (81.9)	188 (85.5)	107 (94.7)	
Time spent to rest, *n* (%)				
*Decreased*	6 (2.6)	2 (0.9)	0 (0.0)	<.001
*Same as before*	105 (46.3)	30 (13.6)	17 (15.0)	
*Increased*	116 (51.1)	188 (85.5)	96 (85.0)	
Time spent to relax, *n* (%)				
*Decreased*	8 (3.5)	8 (3.6)	2 (1.8)	<.001
*Same as before*	90 (39.6)	28 (12.7)	13 (11.5)	
*Increased*	129 (56.8)	184 (83.6)	98 (86.7)	
Time spent to exercise, *n* (%)				
*Decreased*	16 (7.0)	9 (4.1)	18 (15.9)	<.001
*Same as before*	159 (70.0)	194 (88.2)	84 (74.3)	
*Increased*	52 (22.9)	17 (7.7)	11 (9.7)	

### Attitudes towards COVID-19

More than half of pregnant women (57.9%) reported that they knew about the SARS-CoV-2 and relevant prevention knowledge well (Table 4). In addition, majority of pregnant women reported that they were concerned about the COVID-19 progress control (60.0%), COVID-19 pandemic was far away from them (60.9%) and ‘pregnant women were more vulnerable to the COVID-19 pandemic than others’ (78.6%). Pregnant women in first trimester of pregnancy were more likely to agree that ‘pregnant women were more vulnerable to the COVID-19 than others’ than pregnant women in second and third trimesters of pregnancy (*p* < .001).

**Table 4.** Attitudes towards COVID-19 by trimesters of pregnancy.

	Trimesters			*p*-value
	First (*n* = 227)	Second (*n* = 220)	Third (*n* = 113)	
Know SARS-CoV-2 and relevant prevention knowledge well, *n* (%)				
*Yes*	140 (61.7)	123 (56.4)	60 (53.1)	.272
*No*	87 (38.3)	96 (43.6)	53 (46.9)	
Concerned about the COVID-19 progress control, *n* (%)				
*Yes*	140 (61.7)	127 (57.7)	69 (61.1)	.673
*No*	87 (38.3)	93 (42.3)	44 (38.9)	
COVID-19 pandemic is far away from me, *n* (%)				
*Yes*	141 (62.1)	130 (59.1)	70 (61.9)	.781
*No*	86 (37.9)	90 (40.9)	43 (38.1)	
Pregnant women are more vulnerable to the COVID-19 than others, *n* (%)				
*Yes*	196 (86.3)	163 (74.1)	81 (71.7)	<.001
*No*	31 (13.7)	57 (25.9)	32 (28.3)	

### Impact of event scale

The overall mean IES in pregnant women was 31.4 ± 13.7, reflecting moderate-to-severe stressful impact (Table 5). The mean IES of pregnant women in second trimester of pregnancy was the highest (33.8), followed by pregnant women in first and third trimesters of pregnancy (30.1 and 29.3, respectively) (*p* = .016). Overall, there were 67.1% of pregnant women who had an IES value ⩾26. There was a significant association between the percentages of pregnant women with IES ⩾26 and trimester of pregnancy (*p* < .001). Pregnant women in second trimester of pregnancy were more likely to have an IES >26 than pregnant women in first and third trimesters of pregnancy (*p* < .001). The overall mean for intrusion and avoidance of pregnant women were 14.4 ± 7.0 and 17.0 ± 7.4, respectively. The mean intrusion of pregnant women in second trimester of pregnancy was the highest (15.5), followed by pregnant women in first and third trimesters of pregnancy (13.7 and 13.5, respectively) (*p* = .006). In addition, the mean avoidance of pregnant women in second trimester of pregnancy was the highest (18.3), followed by pregnant women in first and third trimesters of pregnancy (16.4 and 15.8, respectively) (*p* = .004).

**Table 5.** Negative health impacts by trimesters of pregnancy.

	Trimesters			*p*-value
	First (*n* = 227)	Second (*n* = 220)	Third (*n* = 113)	
IES	30.1 ± 13.5	33.8 ± 14.2	29.3 ± 13.7	.016
IES >26, *n* (%)	129 (56.8)	175 (79.5)	73 (63.7)	<.001
Increased work stress, *n* (%)				
*Yes*	160 (70.5)	123 (55.9)	69 (61.1)	.006
*No*	67 (29.5)	97 (44.1)	44 (38.9)	
Increased financial stress, *n* (%)				
*Yes*	164 (72.2)	144 (65.5)	80 (70.8)	.276
*No*	63 (27.8)	76 (34.5)	33 (29.2)	
Increased home stress, *n* (%)				
*Yes*	161 (70.9)	112 (50.9)	61 (54.0)	<.001
*No*	66 (29.1)	108 (49.1)	52 (46.0)	
Feel horrified due to the COVID-19, *n* (%)				
*Yes*	141 (62.1)	122 (55.5)	70 (61.9)	.299
*No*	86 (37.9)	98 (44.5)	43 (38.1)	
Feel apprehensive due to the COVID-19, *n* (%)				
*Yes*	147 (64.8)	113 (51.4)	59 (52.2)	.009
*No*	80 (35.2)	107 (48.6)	54 (47.8)	
Feel helpless due to the COVID-19, *n* (%)				
*Yes*	148 (65.2)	164 (74.5)	66 (58.4)	.008
*No*	79 (34.8)	56 (25.5)	47 (41.6)	

### Other indicators of negative mental health impacts

Majority of pregnant women reported increased financial stress (69.3%), increased stress from work (62.9%) and home (59.6%) (Table 5). In addition, more than half of pregnant women reported that they experienced horrified (59.5%), apprehensive (57.0%) and helpless feelings (67.5%) during the early stages of the COVID-19 pandemic. There was a significant association between trimesters of pregnancy and some indicators of negative health impacts (including increased stress from work, increased stress from home, feeling apprehensive and helpless during the early stages of the COVID-19 pandemic) (all *p* < .005). Pregnant women in 1st trimester of pregnancy were more likely to report increased stress from work, increased stress from home and feel apprehensive than pregnant women in second and third trimesters of pregnancy (*p* < .05). On the other hand, pregnant women in second trimester of pregnancy were more likely to feel helpless than pregnant women in first and third trimesters of pregnancy (*p* = .008).

## Discussion

To the best of our knowledge, our study was one of the first studies to assess the mental health, quality of life, attitude, psychological and lifestyle changes among Chinese pregnant women amid the COVID-19 pandemic’s immediate wake. As of July 2020, there have been more than 10 million confirmed cases and >500,000 death worldwide, causing a pandemic within just a few months since the COVID-19 outbreak was initially reported in Wuhan, Hubei province China in December 2019 (WHO, 2020). During pregnancy, women experience some biological adaptive changes which may make them more vulnerable to certain viral infections including severe acute respiratory syndrome (SARS) (Luo & Yin, 2020). In addition, there is limited information regarding the transmission and management of pregnant women positive for COVID-19 (Ali & Shahil Feroz, 2020). Although the prevalence of mental disorders in pregnant women is about 10%, it is suggested that the prevalence of mental disorders, especially anxiety and depression may increase significantly amid the COVID-19 pandemic (Zeng et al., 2020). This is because the current rapid increase in confirmed COVID-19 cases worldwide would certainly exacerbate the stress level among pregnant women, especially those with restricted access to antenatal care service amid the COVID-19 pandemic (Ali & Shahil Feroz, 2020).

Our study reported that the overall mean IES in pregnant women was 31.4 ± 13.7, with 67.1% of pregnant women had an IES ⩾26, indicating moderate-to-severe stressful impact during the early stages of the COVID-19 pandemic. In addition, our study reported that pregnant women in second trimester of pregnancy were more likely to have an IES >26 than pregnant women in first and third trimesters of pregnancy (*p* < .05). Our findings were similar with the results reported in Italian pregnant women by Saccone et al., 2020. The authors reported a moderate-to-severe psychological impact with an overall IES score of 36.9 (Saccone et al., 2020). In addition, another study by Liu et al. reported that the COVID-19 pandemic had aggravated anxiety among pregnant women, especially in Wuhan (Liu et al., 2020). Therefore, the mental health repercussion of the COVID-19 pandemic among pregnant women should not be ignored because it is a major public health issue, which needs timely and appropriate support from the relevant healthcare authorities (Ali & Shahil Feroz, 2020).

In addition, our study reported increased financial stress, increased stress from work and home among pregnant women. It is suggested that pregnant women might also at increased risk of developing mental health issues caused by the external factors including the stress they encountered from work, home and financial issues, especially during this difficult period (Liu et al., 2020). Our study also reported that during the early stages of the COVID-19 pandemic, more than half of pregnant women experienced horrified, apprehensive and helpless feelings, which might be also the symptoms of antennal depression (Humayun et al., 2013). However, the diagnosis of antenatal depression is challenging because it overlaps with the physiological signs of pregnancy (Zeng et al., 2015). Moreover, during the routine antenatal care, the symptoms of antenatal depression might be overlooked by the healthcare providers because they generally concentrate on the physical health aspects of pregnancy (Strass & Billay, 2008). Antenatal depression has been reported to be associated with some negative neonatal outcomes including low appearance, pulse, grimace, activity and respiration (APGAR) score, birth weight and delayed cognitive development (Bonari et al., 2004). Therefore, we recommend that healthcare providers should consider providing some early intervention strategies to pregnant women with depressive symptoms during the COVID-19 pandemic. In addition, some active coping strategies can be promoted to women with a history of mental health disorders (Zeng et al., 2015).

In addition, our study reported that majority of pregnant women had paid more attention to their mental health condition, spent time to rest and relax during the early stages of the COVID-19 pandemic. It is suggested that exposure to stressful impact caused by the COVID-19 pandemic may have reminded pregnant women to pay more attention to mental health and spend more time resting (Liu et al., 2020). However, only a minority of pregnant women, especially pregnant women in first trimester had spent more time exercising. One possible reason might be due to the closure of outdoor parks and recreation centres during the COVID-19 pandemic (Davenport et al., 2020).

Our study reported that 57.9% of pregnant women knew about the SARS-CoV-2 and relevant prevention knowledge well. In addition, majority of pregnant women (78.6%) reported that ‘pregnant women were more vulnerable to the COVID-19 pandemic than others’. It is important to note that during the COVID-19 pandemic, pregnant women will still give birth. However, the lack of information regarding the consequences of the COVID-19 pandemic on pregnant women and their newborns are still not clearly understood. As a result, this can lead to increased stressful level and anxiety among pregnant women, women who are planning to get pregnant and their family members (Davenport et al., 2020). Therefore, some public health measures are urgently needed to mitigate the consequences of COVID-19 pandemic on pregnant women (Liu et al., 2020).

The strengths of our study included the capture of the mental health impact among pregnant women during the early stages of the COVID-19 pandemic. In addition, our study documented the attitudes, lifestyle changes and stressful impact among Chinese pregnant women, which can be used for formulating targeted mental health service by the relevant health authorities. Our findings are also important to provide some information regarding the potentially modifiable factors towards designing targeted public health intervention and education materials for pregnant women. However, one limitation of our study was that this was a cross-sectional study and therefore was not representative of all pregnant women in mainland China. Future studies should consider recruiting more pregnant women and postpartum women from different Chinese provinces using random sampling to overcome the limitations of our present study.

## Conclusion

In conclusion, our study reported moderate-to-severe stressful impact among Chinese pregnant women during the early stages of the COVID-19 pandemic, which highlighted the strong need for heightened assessment of mental health among pregnant women. Although there is a growing understanding of the virology, epidemiology and clinical treatment of COVID-19 patients, the psychology impacts caused by the COVID-19 pandemic should not be neglected. In addition, our study findings, combined with future research, may serve to inform some targeted public health strategies for supporting pregnant women in the current pandemic and other similar future public health crises.
